# Large-scale analysis by SAGE reveals new mechanisms of *v-erbA *oncogene action

**DOI:** 10.1186/1471-2164-8-390

**Published:** 2007-10-26

**Authors:** Corinne Bresson, Celine Keime, Claudine Faure, Yann Letrillard, Maud Barbado, Sandra Sanfilippo, Najate Benhra, Olivier Gandrillon, Sandrine Gonin-Giraud

**Affiliations:** 1 Université de Lyon, Lyon, F-69003, France; 2 CNRS, UMR5534, Centre de génétique moléculaire et cellulaire, Villeurbanne, F-69622, France

## Abstract

**Background:**

The *v-erbA *oncogene, carried by the Avian Erythroblastosis Virus, derives from the *c-erbAα *proto-oncogene that encodes the nuclear receptor for triiodothyronine (T3R). v-ErbA transforms erythroid progenitors *in vitro *by blocking their differentiation, supposedly by interference with T3R and RAR (Retinoic Acid Receptor). However, v-ErbA target genes involved in its transforming activity still remain to be identified.

**Results:**

By using Serial Analysis of Gene Expression (SAGE), we identified 110 genes deregulated by v-ErbA and potentially implicated in the transformation process. Bioinformatic analysis of promoter sequence and transcriptional assays point out a potential role of c-Myb in the v-ErbA effect. Furthermore, grouping of newly identified target genes by function revealed both expected (chromatin/transcription) and unexpected (protein metabolism) functions potentially deregulated by v-ErbA. We then focused our study on 15 of the new v-ErbA target genes and demonstrated by real time PCR that in majority their expression was activated neither by T3, nor RA, nor during differentiation. This was unexpected based upon the previously known role of v-ErbA.

**Conclusion:**

This paper suggests the involvement of a wealth of new unanticipated mechanisms of v-ErbA action.

## Background

The Avian Erythroblastosis Virus (AEV) induces erythroleukemia and sarcomas in chickens [1]. This retrovirus also transforms erythroid progenitors and chicken embryo fibroblasts *in vitro*. The AEV carries two viral oncogenes in its genome, *v-erbA *and *v-erbB*. The expression of v-ErbA alone is sufficient to block differentiation of erythrocytic progenitors *in vitro *and *in vivo *[2,3]. *v-erbA *is derived from the *c-erbA *proto-oncogene, which encodes the alpha form of the nuclear receptor for the thyroid hormone triiodothyronine (T3Rα) [4]. This oncogene is expressed as a gag-v-ErbA fusion protein that binds to the DNA but does not bind the hormone T3 [4].

The most widely accepted hypothesis is that v-ErbA antagonizes ligand-dependent activation by T3R, to silence T3 responsive genes even in the presence of T3 [5-7]. The finding that overexpression of T3R and addition of ligand can overcome the blockade of differentiation induced by v-ErbA supports this assumption [8]. Moreover, it was shown that v-ErbA also inhibits transcription mediated by ligand-activated Retinoic Acid Receptors (RAR) and that this inhibition is also of importance since a non-transforming point mutant for v-ErbA, S61G, is unable to block transcription mediated by RAR [9].

It has therefore been proposed that the v-ErbA oncoprotein represses genes that are normally upregulated by T3 and Retinoic Acid (RA) and that are upregulated during the differentiation process [5,9]. Nevertheless, this hypothesis remains speculative as long as v-ErbA target genes harboring those properties are not found. So far, four v-ErbA target genes have been described: *Carbonic anhydrase II *(*CAII*), *erythrocyte anion transporter *(*Band3*), *integrin α2 subunit *(*VLA2*) and *δ-aminolevulinate synthase *(*ALA-S*) [10-12]. It has been demonstrated only in the case of the *CAII *gene that v-ErbA acts as a dominant negative of T3R by occluding the DNA response element and ablating the T3 mediated activation [13]. In contrast, the expression of *Band3*, *ALA-S *and *VLA2 *genes is not controlled by T3 suggesting that v-ErbA may act independently of T3R [10,11]. This is consistent with the observation that v-ErbA also interferes with pathways controlled by other transcription factors [9,14]. Furthermore, functional studies have clearly demonstrated that the repression of *Band3 *and *CAII *by v-ErbA is not sufficient to account for the differentiation blocking ability of this oncoprotein [15].

Therefore, the precise mechanisms of v-ErbA action remain unclear and the identification of relevant v-ErbA target genes represents a mandatory step in its understanding. We thus decided to use a large-scale transcriptomic approach by SAGE (Serial Analysis of Gene Expression) to identify v-ErbA target genes involved in its transforming activity. One of the most important advantages of this technique is the possibility to obtain a comprehensive view of all the transcripts present in a cell population at a given time point, allowing the discovery of new genes [16]. We have used a cellular model of normal immature erythroid progenitors called T2ECs (TGF-α TGF-β induced erythrocytic cells) [17] which are the natural target cells of v-ErbA. In order to identify v-ErbA target genes responsible for the transformation process induced by v-ErbA, we have compared the transcriptome of T2ECs expressing an oncogenic form of v-ErbA with the transcriptome of T2ECs expressing the S61G mutant of v-ErbA [9,18]. This mutant, which carries a glycine in the DNA binding domain, is equivalent to the *c-erbA *sequence at this position, and is defective in its ability to inhibit differentiation and to induce erythroid transformation [18].

Thus, the comparison between the transcriptome of cells expressing either the transforming form of v-ErbA or the S61G mutant of v-ErbA allowed us to generate a list of 110 differentially expressed genes between these two conditions, 15 of which were observed to be repressed by v-ErbA by real-time PCR on different cell populations. A bioinformatic approach revealed the presence of c-Myb binding sites in the promoters of v-ErbA target genes. The identification of these sites coupled with a functional c-Myb transcriptional assay point out a putative role of c-Myb in the transformation process. Unexpectedly, the global analysis of gene functions reveals that two main cellular functions, protein metabolism and chromatin structure/transcriptional regulation, could be deregulated by v-ErbA. We further show here that the expression of most newly identified v-ErbA target genes is neither under the control of T3 nor RA, nor upregulated during differentiation. In conclusion, we have identified several new v-ErbA target genes unknown up to now and we present evidence that *v-erbA *oncogene action involves new unanticipated mechanisms of action.

## Results

### Potential v-ErbA target genes identified by SAGE analysis

We constructed two SAGE libraries of T2ECs infected either with the XJ12 retrovirus which expresses the oncogenic form of *v-erbA *(VA) or with the S61G retrovirus which expresses a non-transforming form of *v-erbA *(NTVA) in order to identify v-ErbA target genes responsible for transformation. 59391 tags were obtained from the VA library and 37347 tags from the NTVA library. We obtained 5764 different tags appearing more than once in the VA library and 4153 different tags in the NTVA library.

**Figure 1:**
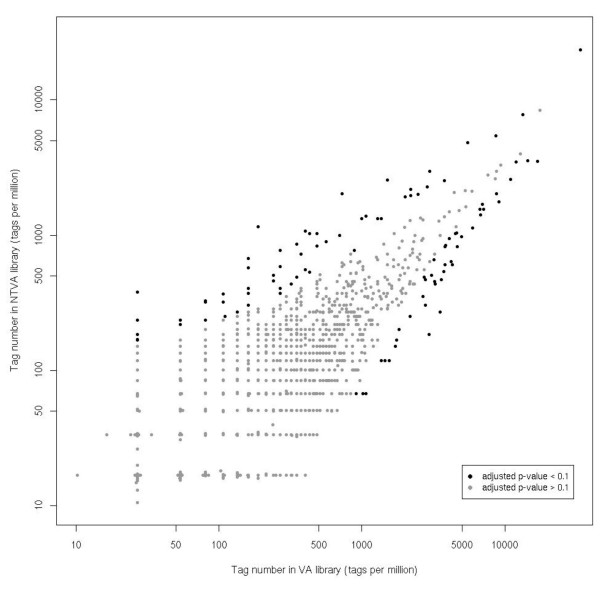
**Comparative gene expression levels between VA and NTVA libraries**. The occurrence number of each tag (normalized in tag per million) in VA (X axis) and NTVA (Y axis) libraries is represented on a logarithmic scale. Each point can represent one or more tags. Colour-coding is based on a statistical analysis (Z test [47]), adjusted for multiple testing according to the method proposed by Benjamini and Hochberg [48]. Black dots represent the 110 tags having an adjusted p-value < 0.1. These tags correspond to genes that are significantly differentially expressed between these 2 conditions. Among these genes, 44 are up-regulated (the corresponding points are located below the first bisecting line) and 66 are down-regulated (the corresponding points are located above the first bisecting line) in T2ECs expressing the transforming form of v-ErbA as compared to T2ECs expressing the non-transforming form of v-ErbA. The gray dots represent all the other tags.

The comparison of these two SAGE libraries revealed 110 differentially expressed genes between these two conditions with a p-value adjusted for multiple testing < 0.1 (Figure 1). 44 of them were up-regulated and 66 down-regulated in T2ECs expressing the transforming form of v-ErbA (VA) as compared to T2ECs expressing the non-transforming form of v-ErbA (NTVA). In view of these results, v-ErbA seems to have mostly a repressive action on gene transcription because it represses more genes than it activates. This essentially repressive action of v-ErbA on gene expression was further confirmed by real-time PCR (see below). However, the vast majority of tags were equally abundant in these two libraries, suggesting that the switch from a normal state toward a pathological state involves a very limited number of genes.

### Presence of c-Myb binding sites in the promoters of v-ErbA repressed target genes

In order to investigate the molecular mechanisms by which v-ErbA regulates gene expression, we next searched for common motifs in the promoters of the v-ErbA target genes identified by SAGE. For that purpose, we first identified SAGE tags by using Identitag [19] and then aligned the corresponding transcripts on the chicken genome. It has been demonstrated for *CAII *that v-ErbA binds to several domains in the proximal promoter sequence, up to 600 bp of the transcription start site (TSS) [20]. Moreover, it has been demonstrated that the position of transcription factor binding sites often lie within a few kb 5' of the basal promoter [21]. We therefore decided to consider as promoter regions 3 kbp 5' of the TSS and 1 kbp 3' of the TSS.

We obtained 31 promoter sequences from genes that appeared as being repressed by the transforming form of v-ErbA by SAGE and 21 sequences from genes that appeared as being activated by the same form of v-ErbA compared to the non transforming form of v-ErbA. We first searched for known v-ErbA response elements [22-25]. All the nuclear receptors of the c-ErbA family recognize derivatives of the same hexameric DNA core motif 5'-AGGTCA-3' [25]. We found response elements based upon this motif only in 3 promoters. The response element PAL-0 (AGGTCATGACCT) is present in *RPL11 *and *Succinate *promoters while the response element DR-4 (AGGTCAnnnnAGGTCA) is present in *RPS3 *and *Succinate *promoters (data not shown). It has been demonstrated that T3R binds preferentially and rather specifically to T3 response element (TRE) of the DR-4 structure [25,26]. The palindromic element without a spacer (PAL-0) is not specific to T3R since it works efficiently as a retinoic acid responsive element (RARE) [25,26]. However, despite the presence of the T3 response element in *RPS3 *promoter, the expression of this gene did not vary in response to T3 (see below). To conclude, out of 52 promoters analyzed, we have only identified 3 promoters with known response element for T3R, RAR and v-ErbA. It is conceivable that the 4 kb region considered is not sufficient since binding sites that would be more distal to the TSS will be missed by this approach. Furthermore, it is also obvious that the simple presence of a transcription factor binding site (TFBS) does not imply an activity of that transcription factor. Finally, even if the protein binds the TFBS, we cannot be informed about the direction of the regulation (i.e. activation or inhibition). Nevertheless, we have found few v-ErbA/T3R/RAR response elements in this region that is in accordance with the experimental assessment of the expression pattern of newly identified v-ErbA target genes under the influence of T3R and RAR (see below).

**Figure 2:**
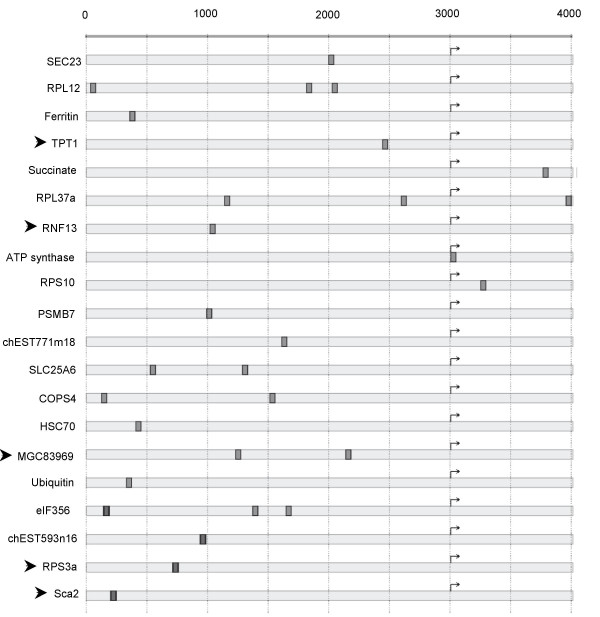
**Position of the c-Myb binding motif in the promoter sequences of v-ErbA target genes**. The position of the c-Myb binding motif (CAGTTA) is represented as grey boxes in the promoter regions of v-ErbA repressed genes containing at least one such motif (i.e. 64% of the v-ErbA repressed genes identified by SAGE). The 4 kb sequence flanking the transcription start site (TSS, shown at position 3000) are displayed. The following parameters were used for extracting this motif: minimal motif size: 5; maximum number of examples in the negative (v-ErbA activated) set: 5; minimum number of examples in the positive (v-ErbA repressed) set: 20. Arrowheads indicate the genes that have been validated as v-ErbA repressed genes by real time PCR.

Since very few v-ErbA target gene promoters contain a known response element for T3R, RAR and v-ErbA, we searched for motifs that could be enriched in these promoters, without any *a priori *on these motifs except its minimal length. For this purpose, we used an implementation of an extended FAVST (Finite Automata-based VST construction) algorithm [27]. This allowed us to identify a c-Myb binding site (CAGTTA) [28] as a signature motif of many newly identified v-ErbA repressed target genes compared with v-ErbA activated target genes. Indeed, 64% of v-ErbA repressed genes have the c-Myb binding motif in their promoter sequences (Figure 2). Among the v-ErbA target genes that contain at least one c-Myb binding site in their promoter region, several have been validated as v-ErbA repressed target genes by real-time RT-PCR (see below). This suggests a potential role for c-Myb in the v-ErbA induced transformation.

**Figure 3:**
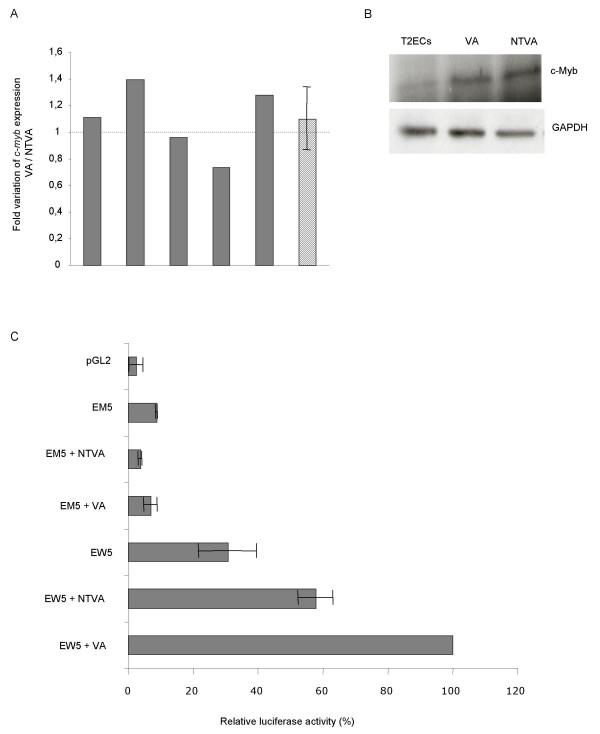
**Expression level of c-Myb in T2ECs expressing v-ErbA or the S61G form of v-ErbA**. 3A – Effect of v-ErbA on the expression level of the *c-myb *gene. Total RNA was extracted from T2ECs expressing either the oncogenic form or the non-transforming form of *v-erbA*. A reverse transcription and real-time PCR analysis were performed to quantify the expression level of *c-myb *gene. The fold variation is represented as VA/NTVA ratio and corresponds to a decrease or an increase of the *c-myb *mRNA in T2ECs expressing the transforming form of v-ErbA (VA) in comparison with T2ECs expressing the non-transforming form of v-ErbA (NTVA). The grey bars represent a mean of ratio calculated using the three reference genes *T-Complex 1*, *hnRNP *and *ATP synthase subunit B1*. The hatched bar is the mean of mRNA accumulation in the five independent experiments. 3B – Effect of v-ErbA on the expression level of the c-Myb protein. T2ECs were either left non-infected (T2ECs), infected by a retrovirus carrying the wild type v-ErbA (VA) or carrying the point-mutated form of v-ErbA, S61G (NTVA). Protein extracts (30 μg) were analyzed and two separate western blots were probed with an anti-c-Myb or anti-GAPDH antibody. 3C – Different effects of v-ErbA and S61G on c-Myb responsive transcription. T2ECs were transfected with reporter plasmids containing either five wild-type (EW5) or mutant (EM5) Myb-binding sites upstream of a simple TATA box. Each reporter was tested with a vector expressing or not the transforming form of v-ErbA (VA) or a vector expressing the S61G mutant for v-ErbA (NTVA) and the β-Galactosidase expressing plasmid. Twenty-four hours after transfection, the cells were analyzed for luciferase activity, which reflects the ability of the c-Myb protein to regulate the gene expression. Luciferase activities were normalized by using β-Gal expression as an internal control. Luciferase activity in the presence of EW5 and the transforming form of v-ErbA was assigned to a value of 100%. The pGL2 plasmid transfected in normal T2ECs represents the negative control (vector which does not contain any binding sites upstream the luciferase coding region). Data are average of two (EM5 and pGL2) to three experiments (EW5) and error bars indicate maximum and minimum values of different sets of data points.

To determine the role of c-Myb in this transformation process, the expression level of this gene was quantified by real-time PCR in T2ECs expressing the transforming form of v-ErbA (VA) compared to T2ECs expressing the non-transforming form of v-ErbA (NTVA) in five independent experiments (Figure 3A). The expression of *c-myb *did not vary between these two conditions. Similarly, we observed that the expression level of the c-Myb protein was also not modified in cells expressing v-ErbA compared with cells expressing the non-transforming S61G mutant (Figure 3B). Although the level of c-Myb protein is increased by both forms of v-ErbA compared with non-infected T2ECs, these results suggest that this upregulation of c-Myb by v-ErbA is not sufficient to induce a transformation process.

Finally, we used a gene reporter assay to test the ability of v-ErbA to transactivate c-Myb. We transfected T2ECs with a reporter plasmid in which five strong c-Myb binding sites were placed upstream of a simple TATA box and a luciferase cDNA [29]. This reporter plasmid (EW5) was clearly active in T2ECs (Figure 3C), which express c-Myb (see above). Moreover, a 1.7-fold increase of luciferase activity was seen with the transforming form of v-ErbA (VA) compared to the S61G mutant for v-ErbA (NTVA). A control reporter gene containing five mutant c-Myb binding sites (EM5) was much less active in these cells, and its activity was unaffected by the different forms of v-ErbA. It is thus conceivable that the c-Myb binding to a real promoter, presenting a higher complexity with several transcription factor binding sites, silencers and enhancers, could result in an inhibition of gene expression controlled by such a promoter. Therefore, although the direction of the functional interaction was unexpected (activation versus repression), these data nevertheless demonstrate that v-ErbA can indeed functionally interact directly or indirectly with the transcriptional activity of endogenous c-Myb in T2ECs.

### v-ErbA mostly affects chromatin/transcription and protein metabolism genes

**Figure 4:**
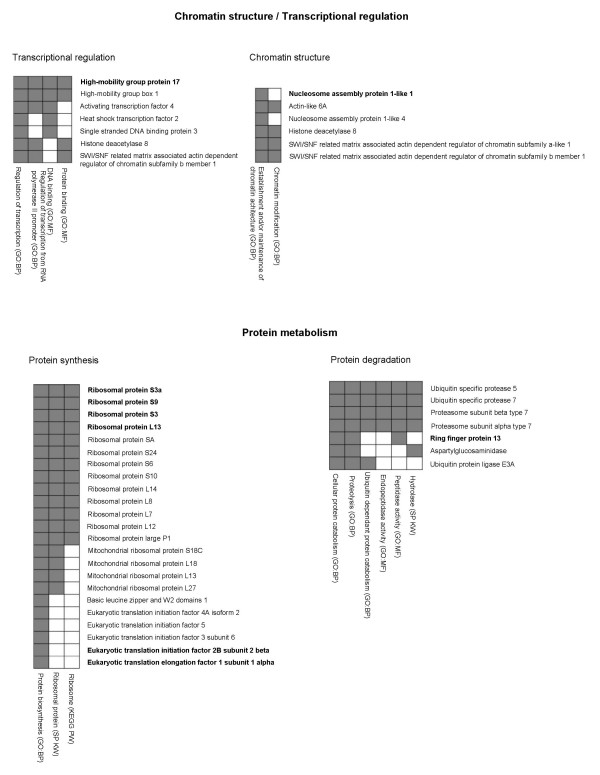
**Cellular functions of genes repressed by the transforming form of v-ErbA**. This figure shows the two main cellular functions of genes repressed by the transforming form of v-ErbA. For each function, the annotations of all genes harbouring this function is represented (only the more precise term in the Gene Ontology and only the annotations concerning more than one half of genes are represented. GO MF: Gene Ontology molecular function, GO BP: Gene Ontology biological process, SP KW: Swissprot keyword, KEGG PW: KEGG pathway). A black square in the intersection of one line (one gene) and one column (one annotation) imply that the corresponding gene has the corresponding annotation. The gene names written in bold correspond to genes for which the differential expression has been confirmed by real-time PCR.

We clustered the newly identified v-ErbA target genes according to the cellular function encoded by their corresponding proteins (Figure 4). Each protein may be implicated in different functions i.e. a protein may belong to different groups.

The use of functional annotations (mainly Gene Ontology (GO) annotations) allowed us to find that many v-ErbA repressed target genes are involved in two main cellular functions: chromatin structure/transcriptional regulation and protein metabolism.

The remodelling of chromatin and transcription regulation constituted a cellular function that was expected to be deregulated by v-ErbA. Indeed, v-ErbA represses transcription via association with corepressors such as NCoR and SMRT [30]. The corepressors, in turn, recruit histone deacetylase (HDAC) containing complexes that stabilize repressive chromatin structure to assure efficient silencing of downstream genes [31,32].

Protein metabolism is the most represented and the most unanticipated category. It suggests the importance of the translation and the protein degradation processes during transformation. Among the genes that are implicated in protein synthesis, we have observed an important number of ribosomal proteins. However, the expression of all ribosomal proteins is not affected by v-ErbA since a vast number of genes encoding ribosomal proteins (*RPL3*, *RPL7*, *RPL12*, *RPL24*, *RPL29*, *RPS4*, *RPS6*, *RPS10 *and *RPS13*) was shown not to vary by real time PCR (data not shown). This suggests that only a specific subset of some ribosomal proteins is affected by v-ErbA.

The other genes down-regulated by v-ErbA, which are not implicated in these two cellular functions, are either implicated in different functions or their functions are unknown. Among these genes, *stem cell antigen 2 *(*sca2*) and *transforming growth factor-β1 *(*TGF-β1*) encode proteins that are involved in the regulation of intracellular signalling pathways. Indeed, we have observed that v-ErbA is able to modulate the TGF-β signalling pathway in T2ECs (Gonin-Giraud et al., submitted). These data suggest a role for v-ErbA in intracellular signalling pathways in agreement with its cytoplasmic localisation [33].

In contrast, the candidate genes whose expression is up-regulated by v-ErbA are fewer and are involved in a greater variety of functions, such as sodium-calcium exchange, chaperone activity, nuclear transport and others.

### 15 v-ErbA target genes validated by real time-PCR on multiple experiments

**Figure 5:**
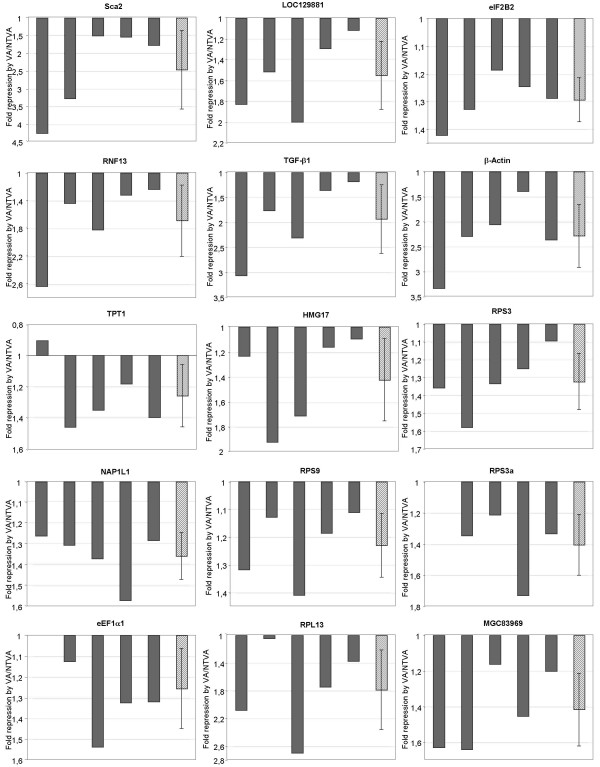
**Real time PCR validation on multiple experiments**. The results of the real-time PCR quantification of genes repressed by the transforming form of v-ErbA and identified by SAGE are presented. Total RNA was extracted from the T2ECs expressing either the oncogenic form or the non-transforming form of *v-erbA*. A reverse transcription and real-time PCR analysis were performed to quantify the expression level of different genes. The fold repression is represented, corresponding to a decrease of mRNA accumulation in T2ECs expressing the transforming form of v-ErbA (VA) in comparison with T2ECs expressing the non-transforming form of v-ErbA (NTVA). The grey bars represent a mean of ratio calculated using three reference genes *T-Complex 1*, *hnRNP *and *ATP synthase subunit B1*. The hatched bar is the mean of mRNA accumulation in the five independent experiments.

The relative expression levels of v-ErbA target genes identified by SAGE were quantified by real-time PCR in T2ECs expressing the transforming form of v-ErbA (VA) compared to T2ECs expressing the non-transforming form of v-ErbA (NTVA) in five independent experiments. We selected 40 candidates and used the sequence of the corresponding transcripts in order to design PCR primers. The differential expression between VA and NTVA conditions has been observed for 15 of them (Figure 5). All these genes are repressed by the transforming form of v-ErbA compared to the non-transforming one. The majority of these genes are involved in protein synthesis, like ribosomal proteins (*RPS3*, *RPS3a*, *RPS9*, *RPL13*) and translation initiation and elongation factors (*eIF2B2*, *eEF1α1*).

We observed that genes were not repressed at the same fold variation in the five different experiments. This can be due to a biological variability between different cultures, as we showed in our previous analysis of the transcriptome of T2ECs in a self-renewal or in a differentiation state [34]. By contrast, the expression of the 25 candidates, which have not been confirmed by real-time PCR, could be explained by different hypothesis (multiple or erroneous identification, variability in gene expression, specific gene variations during the culture that was used for preparing SAGE mRNAs, etc...) as developed in Damiola et al., 2004.

To conclude, out of the 40 candidates tested, we have identified 15 v-ErbA repressed target genes. None of these genes were previously known to be a v-ErbA target gene.

### The expression of the majority of newly identified v-ErbA target genes is not activated nor repressed during differentiation

**Figure 6:**
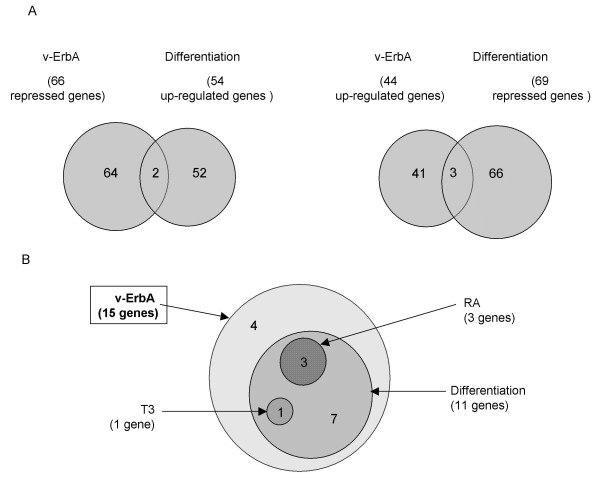
**Assessment of the v-ErbA target gene expression in response to different factors**. 6A – Comparison between the v-ErbA and the differentiation target genes. The Venn Diagrams represent a comparison between the list of v-ErbA repressed target genes (this study) and the genes upregulated during T2ECs differentiation [34] (Left part of the figure) or the list of v-ErbA activated target genes and the genes repressed during T2ECs differentiation (Right part of the figure). 6B – Comparison of v-ErbA, differentiation, T3 and RA target genes. The Venn diagram summarizes the overlap in the sets of genes that were activated or repressed in response to v-ErbA or/and T3 or/and RA or/and during the differentiation process in T2ECs.

In order to test the initial hypothesis that the erythroid progenitors transformation by v-ErbA involves a blockade of the differentiation process, we compared the sets of genes obtained by SAGE analysis in this and in our previous studies [34]. We have identified 2 genes whose expression was found to be increased in T2ECs in a differentiation state versus self-renewal state [34] and decreased in T2ECs expressing v-ErbA versus T2ECs expressing S61G (Figure 6A, left part). On the other hand, we have identified 3 genes whose expression was found to be decreased in T2ECs in a differentiation state versus self-renewal state [34] and increased in T2ECs expressing v-ErbA versus T2ECs expressing S61G (Figure 6A, right part). These 5 genes may be involved both in the T2ECs self-renewal and in the transforming process induced by v-ErbA. Altogether, there is a very modest overlap between these gene lists, suggesting that in majority the v-ErbA target genes might not be deregulated during differentiation, in contrast with our expectations.

We therefore studied by real time PCR the expression of the 15 confirmed v-ErbA target genes during the differentiation process (Table 1 and see Additional File 1). We observed that, among these v-ErbA target genes, some are activated such as *TPT1, RPS9 *and *RPS3a*. This confirms that differentiation related target genes can indeed be inhibited by v-ErbA. However, we observed that the expression of most v-ErbA target genes is either repressed or does not vary during differentiation. These results suggest other mechanisms of v-ErbA action, independent of a mere blockade of differentiation.

**Table 1:** Expression of the v-ErbA target genes in T2ECs during the differentiation process or following a T3, RA or T3 and RA treatment.

Genes	Differentiation	T3	RA	T3+RA
Sca2	- -	0	+ +	+ +
LOC129881	- -	0	+	0
eIF2B2	-	0	+	+
RNF13	-	0	0	0
TGF-β1	- -	-	0	-
β-Actin	- -	0	0	0
TPT1	+	0	0	0
RPS3	0	0	0	0
NAP1L1	-	0	0	0
RPS9	+	0	0	0
RPS3a	+	0	0	0
eEF1α1	0*	0	0	0
RPL13	0	0	0	0
MGC83969	0	0	0	0
HMG17	-	0	0	0

Expression levels of v-ErbA target genes, quantified by real-time PCR, were classified into three groups as follows: (++) strong activation, (+) moderate activation, (0) no variation, (-) repression and (--) strong repression by the different treatments. The table is the synthesis of the results obtained. For actual values and conditions used – see supplementary data. We used, as the reference gene for this experiment, eEF1α1* in agreement with Damiola et al., 2004.

### T3 and RA do not activate the majority of newly identified v-ErbA target genes

The v-ErbA oncoprotein was initially described to be a transcriptional repressor for genes normally activated by T3 Receptor (T3R) and RA Receptor (RAR) [5,9]. Therefore, we have analyzed the expression of v-ErbA target genes by real-time PCR following a treatment by T3 and RA (Table 1 and see Additional File 2). We observed that, among these genes, some are activated by RAR because their expression is induced by RA treatment in normal cells, such as *sca2, LOC129881 *and *eIF2B2*. This confirms that activation by RAR is indeed inhibited by v-ErbA. However, the expression of a vast majority of newly identified v-ErbA target genes is either repressed by T3 or does not vary in response to T3 and RA. These results suggest that v-ErbA must also act by T3R and RAR independent mechanisms in the transformation process.

Moreover, among the 11 differentiation target genes identified, the expression of only 3 genes is activated during the differentiation process and repressed by v-ErbA (Table 1 Figure 6B). To conclude, most of the newly identified v-ErbA target genes are not activated by T3, RA and differentiation.

### The newly identified v-ErbA target genes are cell type specific

**Figure 7:**
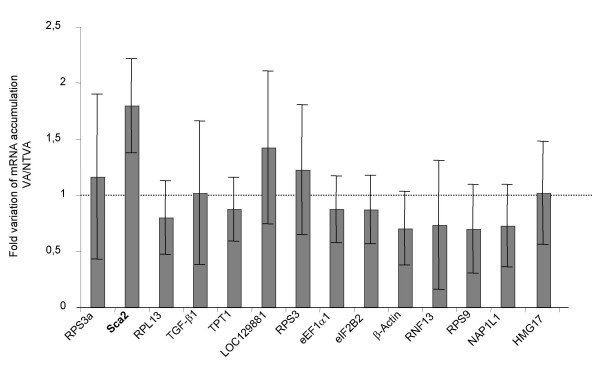
**Expression of the v-ErbA target genes identified in T2ECs, in CEFs expressing v-ErbA or the S61G form of v-ErbA**. Total RNA was extracted from CEFs expressing either the oncogenic form or the non-transforming form of *v-erbA*. A reverse transcription and real-time PCR analysis were performed to quantify the expression level of genes, which are repressed by v-ErbA in T2ECs. The fold variation is represented as VA/NTVA ratio and corresponds to a decrease or an increase of the different mRNA in CEFs expressing the transforming form of v-ErbA (VA) in comparison with CEFs expressing the non-transforming form of v-ErbA (NTVA). The results were normalized using four reference genes *T-Complex 1*, *hnRNP*, *ATP synthase subunit B1 *and *GAPDH*, and correspond to the mean with standard deviation of four to five independent experiments. This figure presents the results of the expression level quantification of most v-ErbA target genes, except for *MGC83969 *gene that could not be amplified.

The v-ErbA oncoprotein was initially demonstrated to be a constitutive repressor of genes activated by T3 in CV-1 cells [5,6] and by T3 and RA in MCF-7 cells [9]. This suggests that the new mechanisms of v-ErbA action highlighted in this paper could be due to the cell type in which its action was studied. In order to determine if the newly identified v-ErbA target genes identified in T2ECs are the same target genes of v-ErbA as in other cells, we have compared them with Chicken Embryo Fibroblasts (CEFs). v-ErbA has been described to alter the growth control of these cells [35]. We observed that the expression of most newly identified v-ErbA target genes was not modulated by v-ErbA in CEFs other than the *sca2 *gene (Figure 7). v-ErbA does not have the same biological effect on different cells (CEFs and T2ECs) and the genes deregulated under its influence are not the same in these two cellular models. These results suggest that the action of the v-ErbA oncoprotein and its target genes depend on the nature of the host cell.

## Discussion

In this paper, we identified new v-ErbA target genes. All of these genes were found to be repressed by v-ErbA and involved in protein metabolism and in chromatin structure/transcriptional regulation. A role for c-Myb in the transformation process induced by v-ErbA could be one of the indirect mechanisms of v-ErbA action. Furthermore, this study clearly illustrates new mechanisms of v-ErbA action, independent from T3R, RAR and erythroid differentiation.

To identify the target genes of the v-ErbA oncoprotein, previous studies have analyzed either the expression of erythroid specific genes [12], of genes which are regulated by T3 [10] or the expression of different membrane antigens [11] in cells transformed by v-ErbA. Thus, v-ErbA has been reported to repress the expression of only four genes: *ALA-S*, *Band3 *[12], *CAII *[10] and *VLA2 *[11]. Repression of *Band3 *and *CAII *by v-ErbA cannot explain altogether the blockade of the differentiation induced by the oncogene, since reexpression of these two proteins had little (CAII) or no effect (Band3) on the differentiation of v-ErbA transformed cells [15]. We searched for these four well-established v-ErbA target genes in the two SAGE libraries of T2ECs expressing either the oncogenic form of v-ErbA (VA) or the non-transforming form of v-ErbA (NTVA). We found only CAII in these libraries. Different reasons can explain this result, including polymorphism in the tag sequence, and a weak expression of the other genes under these two conditions. This suggests that more v-ErbA target genes could be found by a deeper sequencing of SAGE libraries. However, we quantified the expression of these previously identified targets of v-ErbA by real-time PCR in several independent experiments. Only *VLA2 *and *Band3 *are repressed by the transforming form of v-ErbA compared to the non-transforming one (data not shown). These results suggest that these genes could be indeed involved in the transformation process induced by v-ErbA as previously suggested for *Band3 *and *VLA2 *[11,15]. Thus, the identity of cellular genes whose deregulation by the *v-erbA *oncogene is responsible for the transformation remained an open question. By using a global approach by SAGE, we compared the transcriptome of T2ECs expressing the oncogenic form of v-ErbA with the transcriptome of T2ECs expressing the non-transforming form of v-ErbA (S61G). This allowed us to identify several new v-ErbA target genes potentially involved in the transformation induced by the oncogene. The vast majority of expressed genes were not differentially expressed between the two conditions, suggesting that the switch from a normal state toward a pathological state involves a very limited number of genes and small variations.

The newly identified v-ErbA target genes can be either primary or secondary consequences of v-ErbA action. Indeed, v-ErbA target genes involved in the transformation process cannot be differentiated from genes whose expression is modulated consequently to the transformation process. The study of the biological function of each v-ErbA target gene could answer in part this question. Moreover, Genome wide Chromatin Immunoprecipitation (ChIP) assay could be performed to determine the amount of target genes that are susceptible to be under the direct influence of v-ErbA. Among the four previously established target genes of v-ErbA, only *CAII *was demonstrated to be a primary target of v-ErbA [13,20]. The molecular mechanisms through which *ALA-S, Band3 *and *VLA2 *are repressed by v-ErbA remain elusive.

The bioinformatic analysis of the v-ErbA target gene promoter sequences revealed the presence of c-Myb binding sites as a motif that appears more frequently in the promoters of v-ErbA repressed target genes. We observed that v-ErbA upregulates c-Myb expression and that the level of *c-myb *expression was equivalent in cells expressing either wild-type or S61G forms of v-ErbA, as previously demonstrated by Bauer et al. [14]. Since the non-transforming form of v-ErbA also induces c-Myb upregulation, this does not seem to be sufficient *per se *to induce the transformation process. In contrast, reporter gene experiments with a plasmid containing c-Myb binding sites upstream the luciferase gene suggest that v-ErbA, in contrast to S61G, could activate c-Myb by either direct or indirect interactions which will lead to the repression of a subset of key genes. These data propose that a functional interaction with c-Myb could play a role in the transforming process induced by v-ErbA. The *c-myb *proto-oncogene encodes a transcription factor that functions as an activator as well as a repressor of transcription and appears to play a critical role in the regulation of hematopoietic cell differentiation [36]. Its transcription is typically down-regulated during the erythroid cell maturation process [37] and constitutive expression of *c-myb *blocks erythroid differentiation [38].

It has been proposed that v-ErbA represses at least a subset of genes that are normally upregulated by T3 and/or RA and that are possibly linked to the differentiation program of the cell [5,9]. However, our results suggest that v-ErbA could modulate the transcription of a third group of genes that are not under the control of RAR or T3R, or not affected by the differentiation process. The evidence that all TR target genes are not necessary v-ErbA target genes is also supported by DNA binding specificity studies strengthened by functional assays [23,24]. The evidence that some RAR target genes are not v-ErbA target genes is also reinforced by experiments using different retinoic acid response elements and seems to depend upon the cell type tested [9,39-41]. The *v-erbA *oncogene has accumulated mutations that could have created a v-ErbA specific subset of target genes with some TR target genes, some RAR target genes and possibly some target genes for other transcription factors. This could lead to the specific repression of a subset of relevant key genes.

The protein synthesis function is one of the most prominent category of v-ErbA deregulated genes, and one of the most unexpected. We observed a reduction in the expression of several genes encoding proteins involved in protein synthesis, suggesting an important role of variations in translation during transformation. However, the expression of all proteins involved in translation, in particularly ribosomal proteins, is not affected by v-ErbA. It is therefore unlikely that a global change in the amount of ribosomes might result from v-ErbA action. In support of this, several studies have suggested an extraribosomal function of at least some ribosomal proteins [42]. Interestingly, ribosomal proteins, translation initiation and elongation factors have been found to play roles in regulating cell growth and transformation due to such extraribosomal activities [42]. One should also note that variation in the amount of mRNA encoding ribosomal proteins has already been observed to be a common feature in large scale transcriptomic analysis ([43] and references therein).

Altogether, this paper suggests the involvement of a wealth of new unanticipated and possibly indirect mechanisms of v-ErbA action, independently of T3R, RAR and differentiation. The function of the deregulated genes is currently under study in our laboratory.

## Methods

### Cell culture

TGF-β1 and TGF-α were purchased from PeproTech as a lyophilized human recombinant protein and resuspended as previously described [17].

All-trans Retinoic acid was purchased from SIGMA, prepared as a 10^-3 ^M solution in ethanol and subsequently diluted in α-MEM.

3,3',5-Triiodo-L-thyronine (Triiodothyronine) was purchased from SIGMA, prepared as a 10^-3 ^M solution in 0,1N NaOH and subsequently diluted in α-MEM.

T2ECs were generated from SPAFAS white leghorn chickens (PA12 line from INRA, Tours, France) as previously described [17]. These cells were expanded in LMI medium (α-MEM medium, 10% FBS (Fetal Bovine Serum), 10^-3 ^M Hepes, 10^-4 ^M β-mercaptoethanol, 10^-6 ^M dexamethasone, 5 ng/ml TGF-α, 1 ng/ml TGF-β1, penicillin and streptomycin (100 U/ml)).

Differentiating cells were obtained by changing the medium of exponentially growing cells into DM17 medium (α-MEM medium, 10% FBS (Fetal Bovine Serum), 10^-3 ^M Hepes, 10^-4 ^M β-mercaptoethanol, penicillin and streptomycin (100 U/ml), 15% ACS (anemic chicken serum), 10 ng/ml of insulin) [17]. ACS was prepared as previously described [41].

Chicken Embryo Fibroblasts (CEFs) were generated from SPAFAS white leghorn chickens (LD1 line from INRA, Tours, France) as previously described [44]. These cells were expanded in growth medium (Ham's F10 medium, TPB, 5% FBS, 1% NCS (Normal Chicken Serum), 0,1% Sodium Bicarbonate, penicillin and streptomycin (100 U/ml) and 2,5 μg/ml Amphotericin B).

### Viral infections

Viruses were produced by cotransfection of pXJ12 [35] or pS61G [9] with pRAV-1 plasmid on CEFs. pXJ12 coexpresses the Neo^r ^gene and the *v-erbA *oncogene [45] and pS61G coexpresses the Neo^r ^gene and a mutant version of *v-erbA *oncogene [9]. CEFs were infected with viral supernatant XJ12 and S61G previously obtained from CEFs transfected, selected in growth medium containing 0,4 mg/ml G418 (Gibco) for 4 days and then expanded in growth medium.

T2ECs were infected with viral supernatant obtained from CEFs infected and selected for 5 days in the presence of 3 mg/ml G418. The infected cells were purified by centrifugation through a density gradient on Lymphocytes Separation Medium (Eurobio) to get rid of dead cells and debris, and seeded in LMI medium.

### SAGE libraries generation and data analysis

RNA extraction and reverse transcription assay were performed as previously described [41].

We prepared RNA samples from T2ECs infected with XJ12 (VA) or infected with S61G (NTVA). Both cultures were originated from the same cell preparation and divided just before the infection of cells. The infection was validated by PCR verification of *v-erbA *mRNA, and verification of the presence of the mutation S61G before proceeding with the construction of the libraries (data not shown).

SAGE library construction was performed with 10 μg of total RNA, using the I-SAGE™ kit (Invitrogen, Carlsbad, CA, USA) according to the manufacturer's protocol. New PCR primers were redesigned for verification steps on chicken mRNAs. cDNA synthesis verification was performed using chicken Cyclin D1 primers (5'-ACGCTCAGGGACTATACAGG-3' and 5'-GTCTGATGGAGTTGTCGG-3'; AT = 62°C) and chicken Cyclin D2 primers (5'-GTGCTAGTAAGCAACCTTAGGC-3' and 5'-GAACAAGCCTGACTTTCTGTGC-3'; AT = 55°C). NlaIII digestion verification was performed using chicken Cyclin D1 primers and chicken c-rel primers (5'-CTGCTCGAATTCAAGCTTCT-3' and 5'-CCAGTTCTTTAATTACCAAACC-3'; AT = 55°C). Linker ligation and BsmFI digestion verification was performed using the DTP-1 and DTP-2 primers provided with the I-SAGE™ kit together with one Cyclin D2 primer. Concatemer clone sequencing was performed by Genome Express (Meylan, France) and by the Genoscope (Evry, France). Tags were extracted from concatemer sequences by using the R package Sagenhaft [46]. The occurrence number of each tag was computed by taking into account the sequencing errors in concatemer sequences (estimated by the Phred quality score for each base), by using the modelling described in Beissbarth et al., 2004.

Tag identification was performed using Identitag [19] and chicken transcript sequences from the TIGR Gene Index release 10 and from the Refseq databank.

In order to assess the significance of the differential expression of tags between the two libraries, we implemented the Z-test [47] using the R language. We adjusted the resulting p-values for multiple testing by using the method proposed by Benjamini and Hochberg [48] and implemented in the Bioconductor R package multtest.

Because the completion of annotation is higher for human genes than for chicken ones, the classification into functional groups is based on the annotation of human genes orthologous to chicken differentially expressed genes. The classification was made with the DAVID software [49].

### Real-time PCR

Real-Time PCR was performed with a LightCycler system (Roche) or MX3000P Stratagene using the LightCycler FastStart DNA Master SYBR Green I (Roche) according to the manufacturer's instructions. Primer design was carried out using the Primer3 software system [50]. In order to assess the quality and the efficiency of the RT reactions, we analysed the expression of different invariant genes, namely *T-Complex1 *(Sigenae accession number: BBSRC60295473F1.1.3.1), *hnRNP *(Sigenae accession number: AB038230.1.3.153), *ATP Synthase subunit B1 *(GenBank accession number: XM_417993.1), *GAPDH *(GenBank accession number: K01458) and *eEF1α1 *(GenBank accession number: NM_204157.2) (only in differentiation experiments) as internal standards. In order to quantify gene expression, standard curves were generated, for each pair of primers, by dilution series of a sample. The relative expression ratio (R) for each target gene was performed using a previously published mathematical method [51]:

ratio = (E_target_) Δ^CP target(control - sample)^/(E_ref_) ^ΔCP ref(control - sample)^

The reference gene is a house-keeping gene transcript. For the target gene and the reference gene, E is the real time PCR efficiency and ΔCP is the crossing point deviation of control versus sample. Real time PCR efficiencies were calculated according to E = 10^(-1/slope)^. For information about the specific PCR conditions and primers sequences used, please contact the authors.

### Western Blot Analysis

Cells were harvested and washed in cold PBS supplemented with 1% phosphatase inhibitor cocktail 2 (Sigma) and complete mini EDTA-free protease inhibitor cocktail (Roche). The protein concentration was determined by using the Dc protein Assay (BioRad). Cells were then lysed in Laemmli buffer (0,125 M Tris pH6,8, 2% SDS, 10% glycerol, 5% β-mercaptoethanol and 0,1% bromophenol blue) following 5 min at 100°C. 30 μg of proteins were analyzed in a sodium dodecyl sulfate-polyacrylamide gel electrophoresis (SDS-PAGE) (12,5% acrylamide) and blotted on Protean BA85 nitrocellulose membrane. After blockage in 0,1% tween-20 tris-buffered saline (TBS) 5% nonfat milk during 1 hour at room temperature, the membrane was probed with primary antibodies (dilution 1/200 in TBS-0,1%Tween-5% nonfat milk for c-Myb antibody (M-19, Santa Cruz) with an incubation of 4 hours at room temperature; dilution 1/30000 in TBS-0,1%Tween-5% BSA for GAPDH antibody (14C10, Cell signaling) with an incubation of 1 hour at room temperature). The membranes were washed twice in TBS-0,1%Tween before incubation during 1 hour at room temperature with a horseradish-peroxidase-coupled secondary anti-rabbit IgG antibody (dilution 1/7000 in TBS-0,1%Tween-5% nonfat milk for c-Myb analysis or in TBS-0,1%Tween-5% BSA for GAPDH analysis). Proteins were detected using the ECL plus western blotting detection system (Amersham).

### Promoter sequence analysis

The WASHUC1 chicken genome assembly was downloaded from the Ensembl ftp site [52] as of September 2006. Transcripts were aligned on the chicken genome using the BLAT algorithm [53]. The most 5' position of this alignment was used as a putative transcription start site (TSS). 3 kbp 5' of the TSS and 1 kbp 3' of the TSS were extracted and stored as promoter regions. This gave us two sets of promoters: 31 sequences from genes that appeared as being repressed by v-ErbA by SAGE and 21 sequences from genes that appeared as being activated by v-ErbA. We then applied on those two sets of sequences an original algorithm called FAVST (Finite Automata-based VST construction) [27]. Marguerite, an implementation of an extended FAVST algorithm will be described elsewhere (Mitasiunaité, I. et al., in preparation; see also [54]). In contrast with other motif-searching algorithm, Marguerite does not try to find statistically overrepresented motifs, as compared to a random distribution. Marguerite is an exact algorithm that searches exhaustively for regular expression-type patterns that are over-represented in a given set of sequences (positive set) and under-represented in a different but related set of sequences (negative set) without any a priori about these patterns excepted their minimal length. Three parameters are required for running Marguerite: the minimum length of the pattern, the maximum number of examples in the negative set, and the minimum number of examples in the positive set (see legend to figure 2). The identification of the putative transcription factor binding motifs found was done using the PATCH function of the commercial version of TRANSFAC [55].

### Transcriptional activation assay

Transient transfections were performed by nucleofection method (Amaxa Technology). For each nucleofection assay, 10 × 10^6 ^cells were resuspended in 100 μl of nucleofector buffer (Cell line Nucleofector kit V, reference VCA-1003; Amaxa Biosciences) and nucleofected with the T-16 program. The β-galactosidase expressing plasmid (pCMV-β-Gal; 0,5 μg), *luc *reporter plasmids containing either five wild-type (polyA-EW5-LUC; 5 μg) or mutant (poly-A-EM5-LUC; 5 μg) Myb binding sites [29], basic pGL2 (5 μg; Promega), pXJ12 expressing the *v-erbA *oncogene (VA; 5 μg) [35,45] and pS61G expressing a mutant version of *v-erbA *(NTVA; 5 μg) [9,18] were transfected into T2ECs. Twenty-four hours after transfection, the cells were assayed for luciferase activity (Bright-Glo™ Luciferase Assay system; Promega) and β-Gal activity (Gal-screen^® ^System; Applied Biosystems). All transfections were performed two to three times and the pCMV-β-Gal control was used to normalize the transfection experiments. Relative luciferase activities were obtained.

## Authors' contributions

CB performed most of the experiments and drafted the manuscript. CK carried out most of the bioinformatical analysis. CF and SG constructed the libraries. MB performed the transcriptional activation assays. SS and NB participated to the real-time PCR analysis. OG and YL performed promoter sequence analysis. OG and SG conceived the study and participated in its design and coordination. All authors read and approved the final manuscript.

## Supplementary Material

Additional file 1 Gene expression quantification in T2ECs during the differentiation process. The data provided represent the results of the real time PCR quantification of v-ErbA target genes during the T2ECs differentiation. Click here for file

Additional file 2 Gene expression quantification in T2ECs grown in the presence of T3, RA or T3 and RA. The data provided represent the results of the real time PCR quantification of v-ErbA target genes in T2ECs grown in the presence of T3, RA, T3 and RA. Click here for file
