# Supplementary material for: Large-scale analysis by SAGE reveals new mechanisms of v-erbA oncogene action
Source: BMC Genomics. 2007 Oct 26;8:390. doi: 10.1186/1471-2164-8-390 (PMC2194726; doi:10.1186/1471-2164-8-390)
Supplement: Additional file 1 — Gene expression quantification in T2ECs during the differentiation process. The data provided represent the results of the real time PCR quantification of v-ErbA target genes during the T2ECs differentiation. [file 1471-2164-8-390-S1.ppt]

## Slide 1
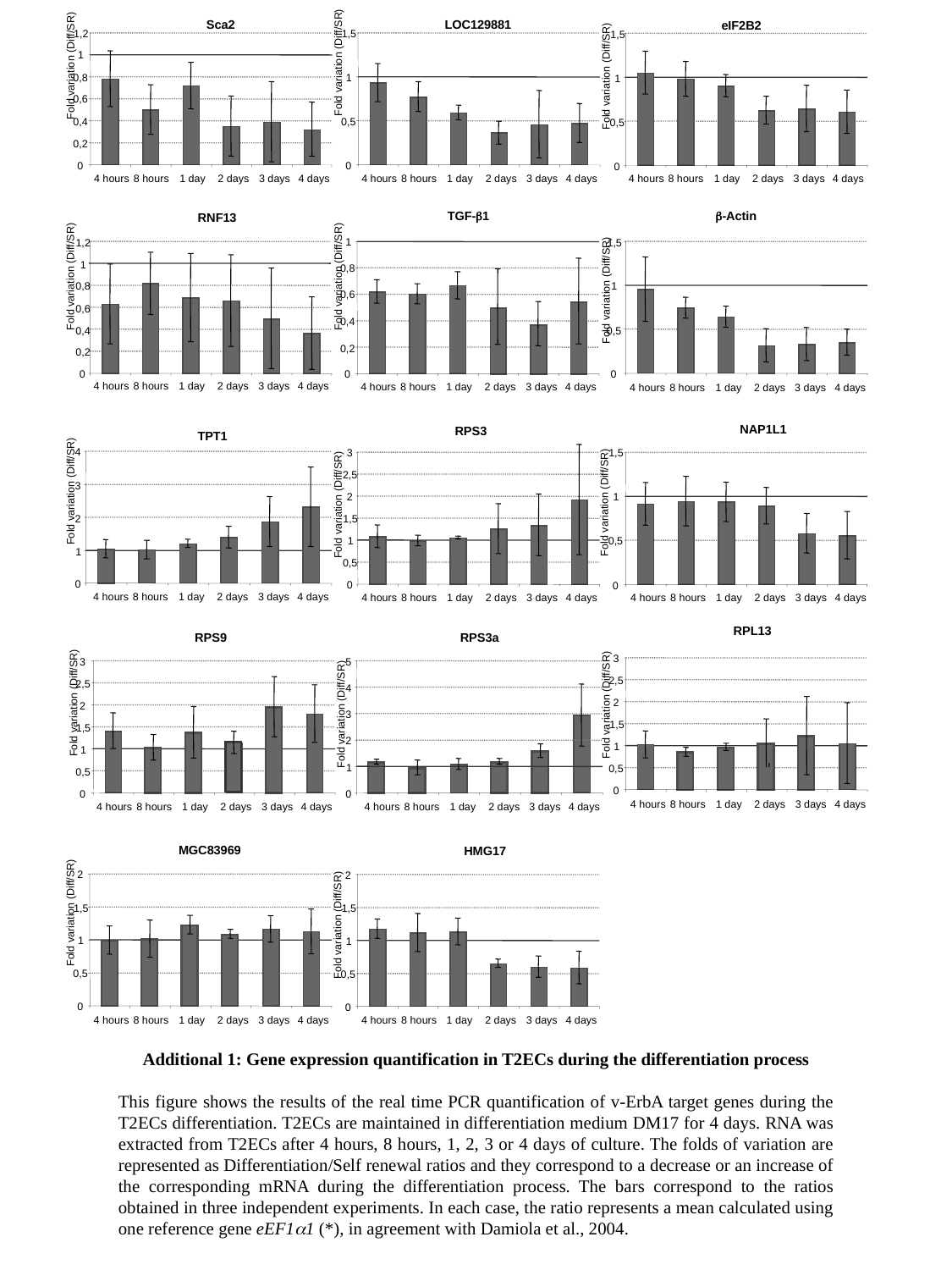

Sca2
LOC129881
eIF2B2
1,2
1,5
1,5
1
Fold variation (Diff/SR)
Fold variation (Diff/SR)
Fold variation (Diff/SR)
0,8
1
1
0,6
0,4
0,5
0,5
0,2
0
0
0
4 hours
8 hours
1 day
2 days
3 days
4 days
4 hours
8 hours
1 day
2 days
3 days
4 days
4 hours
8 hours
1 day
2 days
3 days
4 days
TGF-β1
β-Actin
RNF13
1
1,2
1,5
1
0,8
Fold variation (Diff/SR)
Fold variation (Diff/SR)
0,8
1
Fold variation (Diff/SR)
0,6
0,6
0,4
0,4
0,5
0,2
0,2
0
0
0
4 hours
8 hours
1 day
2 days
3 days
4 days
4 hours
8 hours
1 day
2 days
3 days
4 days
4 hours
8 hours
1 day
2 days
3 days
4 days
NAP1L1
RPS3
TPT1
4
3
1,5
2,5
3
Fold variation (Diff/SR)
2
1
Fold variation (Diff/SR)
Fold variation (Diff/SR)
2
1,5
1
0,5
1
0,5
0
0
0
4 hours
8 hours
1 day
2 days
3 days
4 days
4 hours
8 hours
1 day
2 days
3 days
4 days
4 hours
8 hours
1 day
2 days
3 days
4 days
RPL13
RPS3a
RPS9
3
3
5
2,5
2,5
4
2
Fold variation (Diff/SR)
Fold variation (Diff/SR)
2
3
Fold variation (Diff/SR)
1,5
1,5
2
1
1
1
0,5
0,5
0
0
0
4 hours
8 hours
1 day
2 days
3 days
4 days
4 hours
8 hours
1 day
2 days
3 days
4 days
4 hours
8 hours
1 day
2 days
3 days
4 days
MGC83969
HMG17
2
2
1,5
1,5
Fold variation (Diff/SR)
Fold variation (Diff/SR)
1
1
0,5
0,5
0
0
4 hours
8 hours
1 day
2 days
3 days
4 days
4 hours
8 hours
1 day
2 days
3 days
4 days
Additional 1: Gene expression quantification in T2ECs during the differentiation process
This figure shows the results of the real time PCR quantification of v-ErbA target genes during the T2ECs differentiation. T2ECs are maintained in differentiation medium DM17 for 4 days. RNA was extracted from T2ECs after 4 hours, 8 hours, 1, 2, 3 or 4 days of culture. The folds of variation are represented as Differentiation/Self renewal ratios and they correspond to a decrease or an increase of the corresponding mRNA during the differentiation process. The bars correspond to the ratios obtained in three independent experiments. In each case, the ratio represents a mean calculated using one reference gene eEF1α1 (*), in agreement with Damiola et al., 2004.
