# Supplementary material for: Large-scale analysis by SAGE reveals new mechanisms of v-erbA oncogene action
Source: BMC Genomics. 2007 Oct 26;8:390. doi: 10.1186/1471-2164-8-390 (PMC2194726; doi:10.1186/1471-2164-8-390)
Supplement: Additional file 2 — Gene expression quantification in T2ECs grown in the presence of T3, RA or T3 and RA. The data provided represent the results of the real time PCR quantification of v-ErbA target genes in T2ECs grown in the presence of T3, RA, T3 and RA. [file 1471-2164-8-390-S2.ppt]

## Slide 1
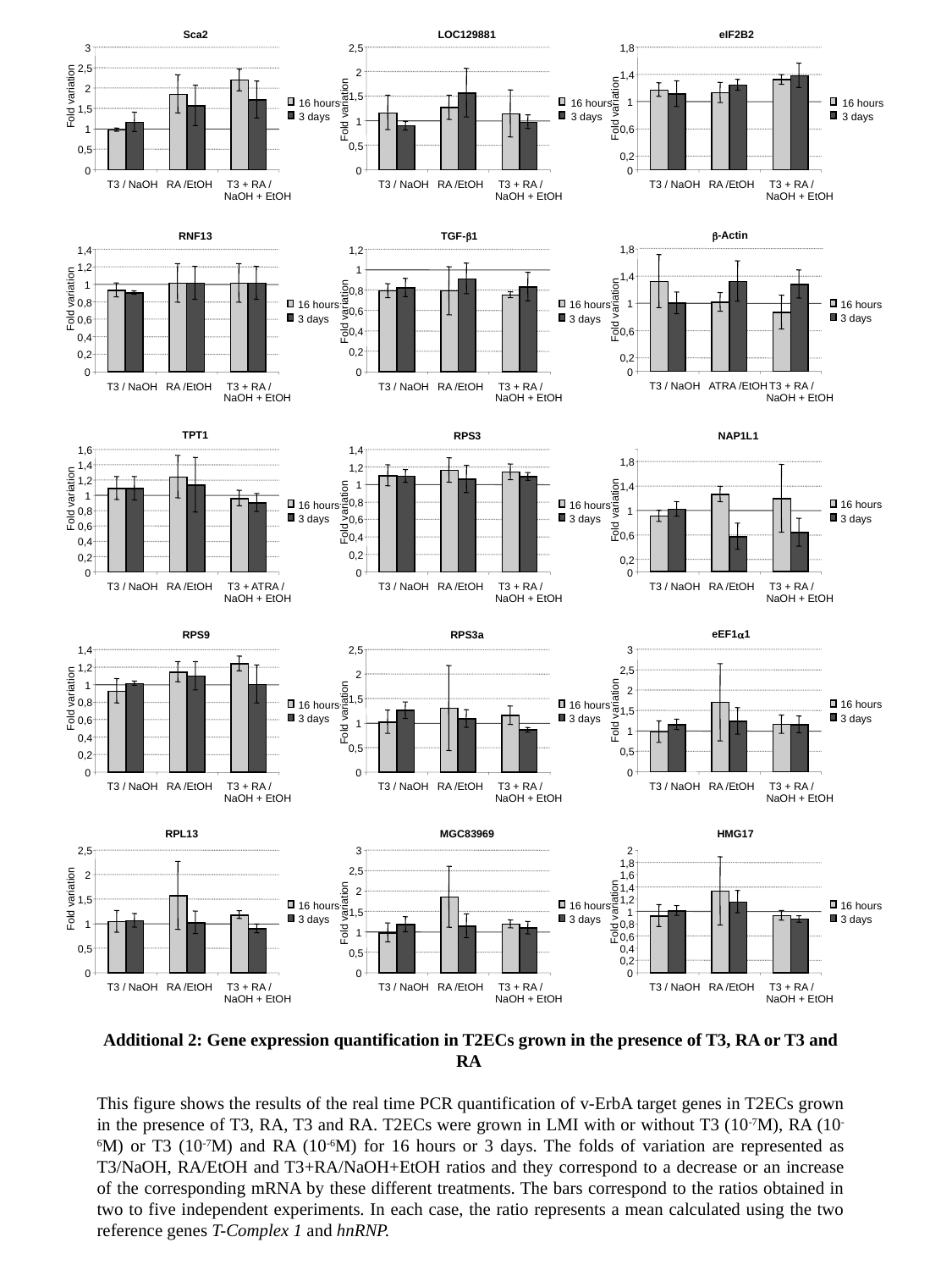

Sca2
LOC129881
eIF2B2
3
2,5
1,8
2,5
2
1,4
2
Fold variation
1,5
1
16 hours
16 hours
16 hours
Fold variation
1,5
Fold variation
3 days
3 days
3 days
1
1
0,6
0,5
0,5
0,2
0
0
0
T3 / NaOH
RA /EtOH
T3 + RA /
T3 / NaOH
RA /EtOH
T3 + RA /
T3 / NaOH
RA /EtOH
T3 + RA /
NaOH + EtOH
NaOH + EtOH
NaOH + EtOH
β-Actin
RNF13
TGF-β1
1,8
1,4
1,2
1,2
1
1,4
1
0,8
Fold variation
0,8
1
16 hours
16 hours
16 hours
Fold variation
0,6
Fold variation
3 days
3 days
3 days
0,6
0,6
0,4
0,4
0,2
0,2
0,2
0
0
0
T3 / NaOH
ATRA /EtOH
T3 + RA /
T3 / NaOH
RA /EtOH
T3 + RA /
T3 / NaOH
RA /EtOH
T3 + RA /
NaOH + EtOH
NaOH + EtOH
NaOH + EtOH
TPT1
NAP1L1
RPS3
1,6
1,4
1,8
1,4
1,2
1,2
1
1,4
1
Fold variation
0,8
16 hours
16 hours
16 hours
Fold variation
0,8
1
Fold variation
3 days
3 days
3 days
0,6
0,6
0,6
0,4
0,4
0,2
0,2
0,2
0
0
0
T3 / NaOH
RA /EtOH
T3 + RA /
T3 / NaOH
RA /EtOH
T3 + ATRA /
T3 / NaOH
RA /EtOH
T3 + RA /
NaOH + EtOH
NaOH + EtOH
NaOH + EtOH
eEF1α1
RPS9
RPS3a
3
1,4
2,5
1,2
2,5
2
1
2
Fold variation
1,5
0,8
16 hours
16 hours
16 hours
Fold variation
1,5
Fold variation
3 days
3 days
3 days
0,6
1
1
0,4
0,5
0,5
0,2
0
0
0
T3 / NaOH
RA /EtOH
T3 + RA /
T3 / NaOH
RA /EtOH
T3 + RA /
T3 / NaOH
RA /EtOH
T3 + RA /
NaOH + EtOH
NaOH + EtOH
NaOH + EtOH
RPL13
MGC83969
HMG17
2,5
3
2
1,8
2,5
2
1,6
1,4
2
Fold variation
1,5
1,2
16 hours
16 hours
16 hours
1,5
1
Fold variation
Fold variation
3 days
3 days
3 days
1
0,8
1
0,6
0,5
0,4
0,5
0,2
0
0
0
T3 / NaOH
RA /EtOH
T3 + RA /
T3 / NaOH
RA /EtOH
T3 + RA /
T3 / NaOH
RA /EtOH
T3 + RA /
NaOH + EtOH
NaOH + EtOH
NaOH + EtOH
Additional 2: Gene expression quantification in T2ECs grown in the presence of T3, RA or T3 and RA
This figure shows the results of the real time PCR quantification of v-ErbA target genes in T2ECs grown in the presence of T3, RA, T3 and RA. T2ECs were grown in LMI with or without T3 (10-7M), RA (10-6M) or T3 (10-7M) and RA (10-6M) for 16 hours or 3 days. The folds of variation are represented as T3/NaOH, RA/EtOH and T3+RA/NaOH+EtOH ratios and they correspond to a decrease or an increase of the corresponding mRNA by these different treatments. The bars correspond to the ratios obtained in two to five independent experiments. In each case, the ratio represents a mean calculated using the two reference genes T-Complex 1 and hnRNP.
